# Nocturnal Hypertension and Attenuated Nocturnal Blood Pressure Dipping is Common in Pediatric Lupus

**DOI:** 10.12688/f1000research.6532.2

**Published:** 2015-11-23

**Authors:** J. Fallon Campbell, Sarah J. Swartz, Scott E. Wenderfer

**Affiliations:** 1 Department of Pediatrics, Renal Section, Baylor College of Medicine, Houston, TX, 77030, USA

**Keywords:** Hypertension, Blood pressure, SLE, Lupus, Pediatric, ABPM, White-coat, Prehypertension

## Abstract

Hypertension is an important manifestation of systemic lupus erythematosus (SLE) but reports of prevalence vary between 20-70% in published reports of adult and pediatric patients. For both children and adults with SLE, the clinical diagnosis and management of hypertension has traditionally been based on guidelines developed for the general population. In clinical trials, the criteria used for defining participants with hypertension are mostly undefined. As a first step towards formally assessing the blood pressure (BP) patterns of children diagnosed with SLE, 24-hr ambulatory BP monitoring data was analyzed on clinic patients who presented with prehypertension or stage I hypertension. In this pediatric SLE cohort (n=10), 20% met daytime criteria for a diagnosis of hypertension. Patterns of BP elevation varied widely with white coat, masked, isolated systolic, and diastolic nocturnal hypertension all identified. Nocturnal hypertension was detected in 60% and attenuated nocturnal BP dipping in 90% of both hypertensive and normotensive SLE patients. In SLE patients, the median nighttime systolic and diastolic loads were 25% and 15.5% compared with median daily loads of 12.5% and 11.5%. Daytime and nighttime systolic and diastolic BP load and nocturnal dipping was compared to a control population consisting of 85 non-SLE patients under 21 years old with prehypertension or stage 1 hypertension presenting to hypertension clinic. Median systolic BP dipped 5.3 mmHg in SLE patients compared to 11.9 mmHg in non-lupus ( *p-value* = 0.001). Median diastolic BP dipped 12.9 mmHg versus 18.5 mmHg in non-lupus ( *p-value* = 0.003). Patterns of BP dysregulation in pediatric SLE merit further exploration. Children with or without SLE displaying prehypertensive or stage 1 casual BP measurements had similar rates of hypertension by ambulatory BP monitoring. However, regardless of BP diagnosis, and independent of kidney involvement, there was an increased proportion with attenuated nocturnal dipping and nocturnal hypertension in SLE patients.

## Introduction

Ambulatory blood pressure monitoring (ABPM) is preferred to casual clinic blood pressure (BP) monitoring in the diagnosis of hypertension (HTN). There are many shortfalls of casual BP readings, including the white coat effect, observer bias/measurement error, regression to the mean with repeated measurements, and variability of blood pressure over time ( Flynn, 2011). Additionally, the published normative values for casual BP are based on the auscultation method, yet many clinic measurements are taken with oscillometric devices ( Woroniecki & Flynn, 2005). Perhaps the most clinically relevant shortfall is the limited outcome data regarding casual BP measurements and end-organ damage or cardiovascular risk.

There is good evidence supporting the utility of ABPM findings in the prediction of cardiovascular outcomes, both in adults and children ( Belsha *et al.*, 1998; Lurbe *et al.*, 2004; Pickering *et al.*, 2006; Singh *et al.*, 2013; Sorof *et al.*, 2002). ABPM can account for the white coat effect as well as measurement and observer errors. Where casual BP measurements account for the magnitude of BP at single points in time, ABPM can define BP loads which measure the proportion of BPs that exceed a defined cutoff, typically the 95 ^th^ percentile as defined by normative data, over a 24-hr period. Therefore ABPM provides a better appreciation of BP trends which can account for the dynamic nature of BP (such as circadian rhythms, nocturnal BP dipping) ( Flynn, 2011).

Although ABPM is a valuable piece of the HTN evaluation, there are potential barriers to its widespread utilization related to both financial and clinical considerations. Insurance companies offer limited reimbursement for ABPM placement and interpretation ( Swartz *et al.*, 2008). Although there is vast evidence for normative values in adults; there is more limited normative data for ABPM interpretation in children. The current normative values are based on approximately 950 healthy children with limited variability in ethnicity/race ( Wühl *et al.*, 2002). Despite these obstacles, ABPM is considered the gold standard for diagnosis of HTN in both adults and pediatrics. It is useful in predicting cardiovascular risk related to HTN but is also helpful in assessing BP in special pediatric populations such as obesity, sickle cell disease, chronic kidney disease, end stage renal disease, and diabetes ( Flynn, 2011). There is limited research on the use of ABPM in the pediatric systemic lupus erythematosus (SLE) population ( Canpolat *et al.*, 2013), though HTN occurs in 20–70% of these patients ( Bogdanovic *et al.*, 2004; Brunner *et al.*, 2002; Lau *et al.*, 2006; Ruggiero *et al.*, 2013). There is also limited data regarding the management of HTN in the SLE population ( Imai *et al.*, 1989; Shaharir *et al.*, 2015), with management traditionally based on guidelines developed for the general population ( Tselios *et al.*, 2014).

Cardiovascular disease is a leading cause of mortality in adults with SLE and though there are many non-traditional risk factors including altered renal function, impaired endothelial function, chronic inflammation, and an activated renin-angiotensin system (RAS) ( Gustafsson *et al.*, 2012; Kiani *et al.*, 2008; Knight & Kaplan, 2013; Pieretti *et al.*, 2007), HTN is still an important risk factor ( Contreras *et al.*, 2005; Ginzler *et al.*, 1993; Petrin *et al.*, 1993; Yang *et al.*, 1994). Therefore the use of standardized definitions to define HTN in SLE patients is crucial in better understanding cardiovascular risk and preventing adverse outcomes. Although isolated nocturnal HTN is not considered sufficient for a diagnosis for systemic hypertension in the general population, it is known to associate with increased risk of cardiac outcomes ( Yee, 2015). Additionally, attenuated nocturnal dipping, even in the setting of normal 24-hr BP, was noted to be an independent predictor of cardiovascular mortality in a large prospective cohort study in Japan ( Ohkubo *et al.*, 2002). Hence, it is also important to use ABPMs to characterize blood pressure patterns in SLE patients so that specific guidelines for ABPM interpretation can be established for this population.

## Methods

### Study population

BP patterns of the 10 SLE study participants (demographics summarized in Table 1) recruited from a single center were retrospectively reviewed using data from 24-hr ambulatory BP monitoring tests performed between February 2012 and April 2013. ABPM was routinely ordered only on SLE patients seen in a multispecialty pediatric lupus clinic who were not currently being treated for active lupus kidney disease (non-renal lupus or nephritis in remission) and with casual BP measurements in prehypertensive or stage 1 hypertensive range ( National High BP Education Program Working Group on High BP in Children and Adolescents, 2004). The inclusion criteria for the SLE cohort were: (1) diagnosis of SLE by American College of Rheumatology (ACR) criteria, (2) age < 21 years, and (3) ABPM performed. There were 85 patients in the non-SLE cohort (43% female, race and ethnicity unknown). The inclusion criteria for the non-SLE cohort included: (1) age < 21 years and (2) ABPM performed. Exclusion criteria for both cohorts: (1) ABPM uninterpretable due to incomplete/missing data, (2) casual BP measurements all < 90th or all > 99th percentile for age, gender, and height, +5 mmHg (3) end stage renal disease, or (4) kidney transplant recipient. The mean age at ABPM for the non-SLE cohort was 12.4 ± 0.4 years, and the mean BMI was 25.1 ± 0.8 kg/m ^2^ using the Mosteller formula. The study protocol was reviewed and approved by the Institutional Review Board for Baylor College of Medicine (H-32061).

**Table 1.** Pediatric SLE Cohort Demographics.

Patient Characteristics	Values (n=10)
Age at ABPM (years)	14.6 ± 0.3
Age at SLE Diagnosis (years)	12.4 ± 1.0
BMI (kg/m ^2^)	25.1 ± 1.1
SLE ACR criteria met	5.8 ± 0.4
Gender (% female)	9 (90)
Race	
African American (%)	4 (40)
Hispanic (%)	3 (30)
Caucasian (%)	3 (30)
	
**Laboratory Findings (within 3 months** **of ABPM)**	
eGFR (ml/min/1.73 m ^2^)	133 ± 6.2
C3 (mg/dL)	83.4 ± 9.5
Sed Rate (mm/hr)	58.7 ± 16.2
Hgb (g/dL)	11.8 ± 0.5
Elevated CRP (%)	1 (10)
Proteinuria (%)	2 (20)
Positive ANA (%)	10 (100)
Positive Anti-DS DNA Antibody (%)	9 (90)
Positive Anti-phospholipid Antibody (%)	9 (90)
	
**Medications (at time of ABPM)**	
Prednisone dose (mg/kg/day)	0.313 ± 0.07
IV corticosteroids (%)	3 (30)
Hydroxychloroquine (%)	9 (90)
Mycophenolate mofetile (%)	3 (30)
Methotrexate (%)	1 (10)
Azathioprine (%)	1 (10)
Aspirin (%)	7 (70)
ACE inhibitor (%)	1 (10)

***Participants.*** As this study was a retrospective chart review, there were no dropouts.

***Sample size.*** As this was a pilot study, the sample size was not determined by formal power analysis. There were 11 consecutive SLE patients and 100 consecutive non-SLE patients with ABPM data identified, but 1 and 15 patients, respectively did not qualify based on the inclusion and exclusion criteria.

***Blinding.*** All interpretation of ABPM data was performed at the time of clinical testing, prior to inception of the study. There was no formal blinding of ABPM data during the data analysis or sensitivity analysis phases.

### Ambulatory Blood Pressure Monitoring

24-hr ABPM was performed using SpaceLabs 90217-1Q or 90217A-1 equipment. SpaceLabs Medical Software (version 90219) was used to evaluate the BP patterns. BP measurements were automatically measured every 20 minutes during the daytime and every 30 minutes during the nighttime over a 24-hr period. Mean diastolic and systolic BPs were calculated for both daytime and nighttime periods and compared to normative data for mean BPs based on age and gender ( Wühl *et al.*, 2002). BP loads were calculated for both diastolic and systolic BP, reflecting the percentage of BP measurements above the 95 ^th^ percentile for gender and age. Blood pressure loads >25% were considered abnormal ( Urbina *et al.*, 2008). Additionally, nocturnal dipping of BP was defined as the difference between daytime and nighttime BPs. Nocturnal dipping <10% was considered abnormal ( Urbina *et al.*, 2008). American Heart Association (AHA) definitions were used to define normal blood pressure, masked, white-coat and sustained HTN ( Flynn, 2011).

### Clinical data

Demographic and clinical data was collected from medical records for the SLE cohort. Demographic data included race and age at diagnosis. Clinical data (from within 3 months of ABPM date) included height, weight, BMI, laboratory results, eGFR using the Schwartz formula ( Schwartz *et al.*, 2009), presence of proteinuria, medications at the time of ABPM, echocardiogram findings including left ventricular hypertrophy (LVH), left ventricular mass index (LVMI), and relative wall thickness (RWT), ACR criteria for SLE, and SLEDAI score (Systemic Lupus Erythematosus Disease Activity Index). Demographic information for the non-SLE cohort was obtained from the SpaceLabs software, including age at the time of ABPM, gender, height, and weight.

### Statistical analysis

Statistical analysis was performed using SigmaPlot software (version 11.0). Patient characteristics and ABPM findings were analyzed using descriptive statistics (medians, and intra-quartile ranges). Fisher exact and Wilcoxon rank sum tests were used to characterize patient demographics and BP patterns. Statistical significance was defined as *p*-value ≤0.05 (two tailed).

## Results

### Patient and clinical characteristics

The study population consisted of ten patients, all of whom met ACR diagnostic criteria for SLE (Patient demographics in Table 1). Whereas 60% had a history of lupus nephritis, none were currently being treated for active kidney disease. At the time of ABPM, mean age of the SLE cohort was 14.6 years. The mean BMI was 25.1 kg/m ^2^. Nine patients were female. Three SLE patients were Hispanic, three were Caucasian, and four were African American. The non-SLE control population consisted of 85 age- and BMI-matched pediatric patients with casual BP measurements between 90–99 ^th^ percentile without kidney disease or diabetes.

Raw ABPM Data for pSLE Cohort File contains the coded ambulatory blood pressure monitoring data for the pediatric SLE cohort abstracted from Space Labs software, using the default 95th percentile cutoff to distinguish normal versus high BP values. ABPM data was matched to demographic, clinical, and laboratory data abstracted from the electronic medical record. N/A (not available) indicates that data was sought but testing was not performed. BMI = body mass index, eGFR = estimated glomerular filtration rate, Hgb = hemoglobin, ESR = erythrocyte sedimentation rate, CRP = Creactive protein, LDL = low density lipoprotein, HDL = high density lipoprotein, ANA = anti-nuclear antibody, AZA = azathioprine, MTX = methotrexate, RTX = rituximab, ACEI = Angiotensin-converting enzyme inhibitor, ARB = angiotensin receptor blocker, LVH = left bentricular hypertrophy, LVMI = left ventricular mass index, RWT = right wall thickness, OSA = obstructive sleep apnea (Dataset 1: Campbell *et al.*, 2015a). Click here for additional data file. Copyright: © 2015 Campbell JF et al. 2015 Data associated with the article are available under the terms of the Creative Commons Zero "No rights reserved" data waiver (CC0 1.0 Public domain dedication).

Raw ABPM Data for non-SLE Cohort File contains the coded ambulatory blood pressure monitoring data and matched demographic data for the non-SLE pediatric cohort abstracted from Space Labs software, using the default 95th percentile cutoff to distinguish normal versus high BP values. Age is in years, BMI = body mass index (Dataset 2: Campbell *et al.*, 2015b). Click here for additional data file. Copyright: © 2015 Campbell JF et al. 2015 Data associated with the article are available under the terms of the Creative Commons Zero "No rights reserved" data waiver (CC0 1.0 Public domain dedication).

All of the SLE patients were antinuclear antibody (ANA) positive, nine were anti-double stranded DNA (anti-dsDNA) antibody positive, and nine tested positive for anti-phospholipid antibodies (aPL). Three SLE patients had echocardiograms and none had sonographic evidence of left ventricular hypertrophy based on adult criteria (LVMI 35.3, 39.2, and 42.9 g/m^2.7, with RWT of 0.40, 0.52, and 0.39). Two patients had proteinuria based on the SLE ACR definition (>0.5 g/day) at the time of ABPM. Among the seven patients with lab values available, the mean ESR was elevated at 58.71 mm/hr (normal <20) and the mean C3 was slightly low at 83.4 mg/dL (normal 90–200).

All SLE patients were prescribed prednisone at the time of ABPM with a mean dose of 0.31 ± 0.08 mg/kg/day. Three patients also received intravenous (IV) pulse steroids within the 3 months prior to ABPM. Two received weekly doses of IV solumedrol (30mg/kg/dose) and the third received a one-time dose in the week prior to ABPM placement. At the time of ABPM, only one was prescribed an ACE-inhibitor, and none were prescribed diuretics or any other anti-hypertensive. No one was treated with rituximab within the 2 years prior to the ABPM, though one patient did receive Rituximab following ABPM. The SLE patients met a median of six SLE ACR criteria for diagnosis of SLE. Five patients met criteria for malar rash; three met criteria for photosensitivity; five had mouth sores; three experienced serositis; eight had arthritis; six had renal involvement; seven met hematologic criteria; nine met immunologic criteria; and one met neurologic criteria. No patients were noted with a documented discoid rash.

### ABPM findings

**Figure 1.**
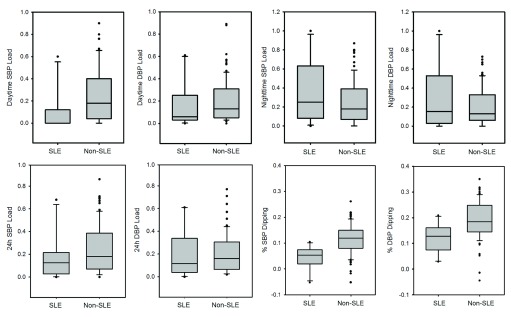
Attenuated BP dipping and trends toward less daytime and more nighttime HTN in pediatric SLE. Box plots indicate median, 10th, 25th, 75th, and 90th percentile data, based on Wilcoxon Analysis. Dots indicate the outliers. Differences in %SBP dipping ( *p* = 0.001) and %DBP dipping ( *p* = 0.003) and daytime SBP load ( *p* = 0.01) were statistically significant whereas differences in nighttime SBP load ( *p* = 0.36) and DBP load ( *p* = 0.59) failed to reach significance.

SLE patients tended to have lower daytime systolic blood pressure (SBP) and diastolic blood pressure (DBP) loads and higher nighttime systolic BP loads as compared to the non-SLE patients, and the decreased median SBP load was statistically significant ( Table 2). Nighttime SBP and DBP loads were higher in SLE patients with a history of nephritis than in non-renal lupus patients, even though the nephritis was in remission at time of ABPM. The SLE cohort also showed a significantly higher rate of attenuated nocturnal dipping in both SBP and DBP, when compared to the non-SLE cohort ( Figure 1). Ninety percent of SLE patients had attenuated nocturnal dipping compared to only 26% of non-SLE patients. SLE patients also had a higher rate of nocturnal HTN, whether in isolation or in conjunction with daytime HTN.

**Table 2.** Higher rates of Attenuated Systolic and Diastolic BP Dipping and Nocturnal Hypertension in Children with SLE.

Median Load * (%)	SLE	Non-SLE	*p-*value	*Non-renal lupus*	*LN in remission*
**24 hr SBP**	12.5	18	0.22	2	20
**24 hr DBP**	11.5	16	0.45	5	18
**Daytime SBP**	0	18	0.01	0	11
**Daytime DBP**	6	13	0.25	3	12
**Nighttime SBP**	25	18	0.36	8	50
**Nighttime DBP**	15.5	13	0.59	8	34
					
Median BP Dipping (%)	SLE	Non-SLE	*p*-value	*Non-renal lupus*	*LN in remission*
**Systolic**	5.3	11.9	0.001	7	3
**Diastolic**	12.9	18.5	0.003	14	11
					
BP Diagnosis * (%)	SLE	Non-SLE	*p*-value	*Non-renal lupus*	*LN in remission*
**Daytime and Nighttime Hypertension**	20	29	0.49	25	17
**Daytime Hypertension ( ± nighttime)**	20	42	0.10	25	17
**Daytime Hypertension only**	0	17	0.20	0	0
**Nighttime Hypertension (± daytime)**	60	39	0.51	25	83
**Nighttime Hypertension only**	40	21	0.45	0	67
**Normal Daytime and Nighttime BP**	40	30	0.74	75	17
**Attenuated Nocturnal Dipping**	90	26	< 0.001	100	83

* based on definition of elevated BP as exceeding the 95th%tile for age and gender (Wuhl, 2002)

Specifically, only two SLE patients met ABPM criteria for both daytime and nighttime HTN; however four additional patients had isolated nocturnal HTN with normal daytime BPs. All of these patients had a history of nephritis. Nine of the SLE patients had attenuated nocturnal dipping, regardless of HTN diagnosis. Of the nine SLE patients with attenuated nocturnal dipping, two had proteinuria at the time of ABPM. The one patient with normal dipping had no proteinuria.

### Relationships between ABPM findings and clinical characteristics

There were no statistically significant associations between most laboratory measures (complement 3 (C3), ANA, anti-dsDNA antibodies, aPL antibodies) and nocturnal HTN or attenuated nocturnal dipping. The two patients without aPL antibodies did not have nocturnal HTN, though they did have attenuated dipping ( Table 3). The one patient who had normal nocturnal dipping was African American, had the highest BMI, low C3 levels, a SLEDAI score of 4, received a dose of pulse steroid within 3 weeks of ABPM, and met more than six SLE ACR criteria. She did have nocturnal and masked HTN. There were no obvious associations between ABPM findings and the presence of specific historical ACR criteria for SLE; however, this study is underpowered to perform formal statistical analysis. Of the six SLE patients who historically met diagnostic criteria for kidney disease, five had nocturnal HTN, while only one of the four patients without a history of nephritis had nocturnal HTN. Moreover, five of the six with a history of nephritis and all four of the non-renal SLE patients had attenuated nocturnal dipping.

**Table 3.** Individual ABPM and Clinical Laboratory Data for Pediatric SLE Cohort *.

#	BP Diagnosis	Age at ABPM	Wake SBP load	Wake DBP load	Sleep SBP load	Sleep DBP load	SBP Dip	DBP Dip	Attenuated Dipping	Nocturnal HTN	BMI	eGFR	C3	DNA Ab	aPL Ab	SLEDAI Score	# ACR criteria met
1	HTN	14	60%	50%	100%	100%	2%	5%	Yes	Yes	22	153	47	Yes	Yes	15	8
2	masked HTN	14	0%	61%	12%	62%	8%	16%	Yes	Yes	22	169	106	Yes	Yes	4	7
3	normotension	17	12%	8%	36%	0%	10%	21%	No	Yes	31	114	44	Yes	Yes	4	6
4	normotension	15	9%	15%	35%	23%	6%	13%	Yes	Yes	25	132	126	Yes	Yes	4	6
5	normotension	15	0%	3%	64%	50%	-5%	3%	Yes	Yes	28	109	50	Yes	Yes	10	6
6	normotension	15	0%	3%	15%	4%	1%	15%	Yes	No	29	137	90	Yes	No	4	5
7	normotension	14	0%	0%	0%	0%	6%	17%	Yes	No	20	131	75	Yes	Yes	8	6
8	normotension	14	12%	17%	63%	44%	3%	8%	Yes	Yes	25	114	86	Yes	Yes	8	6
10	normotension	15	0%	3%	8%	8%	5%	13%	Yes	No	24	142	104	No	No	6	4
9	white coat	13	0%	4%	8%	8%	7%	11%	Yes	No	26	129	106	Yes	Yes	4	4

* based on definition of elevated BP as exceeding the 95th%tile for age and gender (Wuhl, 2002)

There was also no significant difference in attenuated nocturnal dipping between SLE patients who received pulse corticosteroids within 3 months of ABPM and those who had not. Of the three patients who received pulse steroids, two had nocturnal HTN and one did not. Finally, there were no statistically significant association between use of specific immunosuppressive medication usage (azathioprine, mycophenolate mofetile, hydroxychloroquine, methotrexate) and either nocturnal HTN or attenuated nocturnal dipping. The patient who was on an ACE inhibitor at the time of ABPM did have attenuated nocturnal dipping and nocturnal HTN.

Attenuated dipping was not associated with disease duration. The patient with the longest SLE vintage (9 years from SLE diagnosis to time of ABPM) had both nocturnal HTN and attenuated nocturnal dipping, but the three patients with the shortest disease duration (<1 year from SLE diagnosis to time of ABPM) all had attenuated dipping.

The two SLE patients with daytime HTN also had nocturnal HTN and attenuated nocturnal dipping. They were both African American and one was the only male in the cohort. One patient’s disease duration was 9 years whereas the other was diagnosed in the past year. The patient with disease duration of < 1 year was on a higher oral steroid dose and had proteinuria and low C3 level. They both met more than six ACR diagnostic criteria.

All four of the patients with normal nocturnal BPs still had attenuated nocturnal dipping. Three of these patients’ disease duration was ≤ 1 year while the fourth was 6 years. Their SLEDAI scores ranged from 4–8 at the time of ABPM ± 10 days.

### Sensitivity analysis

**Figure 2.**
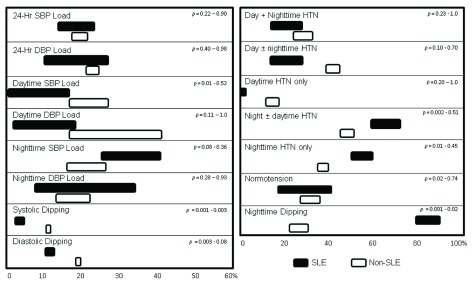
No major effects of methods for ABPM interpretation on results in SLE cohort. Boxes represent the range of medians (left) or means (right) obtained from sensitivity analysis. Analyses were repeated comparing non-SLE to SLE cohort, using either 90th or 95th percentiles, and 25% or 30% load, in the definition for hypertension, and by restricting dataset to only include ABPM findings when success rates of measurements were >75%, when >50 total successful measurements were recorded, or both. Ranges of *p*-values are indicated.

To determine whether disease-specific BP parameters might be influenced by the thresholds used to define hypertension during analysis of the ABPM data, a sensitivity analysis was performed. Since all patients in our pediatric SLE cohort lacked active nephritis and heart disease, a 95% cutoff was used to distinguish normal versus high BP. To test whether using a 90% cutoff would alter the results, all SLE patient ABPM data was re-interpreted. The data was also reanalyzed using BP loads of >30% (per institutional protocol) rather than 25% ( Urbina *et al.*, 2008) to define HTN. In addition, to test if the quality of the ABPM data affected the findings, the comparison between cohorts was repeated after ABPM tests were discarded if either <75% of attempted BP measurements were successful, <50 total measurements were successful, or both. Finally, since 90% of the SLE cohort was female, comparisons were made to the non-SLE controls after eliminating ABPM data from males in the non-SLE cohort. Results showed that decrease in prevalence of daytime SBP load in the SLE cohort lost significance using a 90% cutoff, whereas the increase in incidence of nocturnal HTN became significant using BP loads >30% to define hypertension ( Figure 2). The attenuation of nighttime BP dipping in the SLE cohort and all other ABPM findings were not significantly altered by any of the changes.

Raw Data: Sensitivity Analysis: non-SLE greater than 50 successful readings File contains the coded ambulatory blood pressure monitoring data and matched demographic data for the subset of the non-SLE pediatric cohort meeting a more stringent criterion of ABPM data quality, in particular the successful completion of 50 or more BP measurements within the 24-hour monitoring period. ABPM data was abstracted from Space Labs software, using a 90th percentile cutoff to distinguish normal versus high BP values. Age is in years, BMI = body mass index (Dataset 3: Campbell *et al.*, 2015c). Click here for additional data file. Copyright: © 2015 Campbell JF et al. 2015 Data associated with the article are available under the terms of the Creative Commons Zero "No rights reserved" data waiver (CC0 1.0 Public domain dedication).

Raw Data: Sensitivity Analysis: non-SLE, greater than 75 percent successful File contains the coded ambulatory blood pressure monitoring data and matched demographic data for the subset of the pediatric SLE cohort meeting a more stringent criterion of ABPM data quality, in particular the successful completion of 75% or greater of the total attempted BP measurements within the 24-hour monitoring period. ABPM data was abstracted from Space Labs software, using a 90th percentile cutoff to distinguish normal versus high BP values. Age is in years, BMI = body mass index (Dataset 4: Campbell *et al.*, 2015d). Click here for additional data file. Copyright: © 2015 Campbell JF et al. 2015 Data associated with the article are available under the terms of the Creative Commons Zero "No rights reserved" data waiver (CC0 1.0 Public domain dedication).

Raw Data: Sensitivity Analysis: pSLE greater than 50 successful readings File contains the coded ambulatory blood pressure monitoring data and matched demographic data for the subset of the pediatric SLE cohort meeting a more stringent criterion of ABPM data quality, in particular the successful completion of 50 or more BP measurements within the 24-hour monitoring period. ABPM data was abstracted from Space Labs software, using a 90th percentile cutoff to distinguish normal versus high BP values. Age is in years, BMI = body mass index (Dataset 5: Campbell *et al.*, 2015e). Click here for additional data file. Copyright: © 2015 Campbell JF et al. 2015 Data associated with the article are available under the terms of the Creative Commons Zero "No rights reserved" data waiver (CC0 1.0 Public domain dedication).

Raw Data: Sensitivity Analysis: pSLE greater than 75 percent successful File contains the coded ambulatory blood pressure monitoring data and matched demographic data for the subset of the pediatric SLE cohort meeting a more stringent criterion of ABPM data quality, in particular the successful completion of 75% or greater of the total attempted BP measurements within the 24-hour monitoring period. ABPM data was abstracted from Space Labs software, using a 90th percentile cutoff to distinguish normal versus high BP values. Age is in years, BMI = body mass index (Dataset 6: Campbell *et al.*, 2015f). Click here for additional data file. Copyright: © 2015 Campbell JF et al. 2015 Data associated with the article are available under the terms of the Creative Commons Zero "No rights reserved" data waiver (CC0 1.0 Public domain dedication).

Raw Data: Sensitivity Analysis: pSLE using 90th percentile File contains the coded ambulatory blood pressure monitoring data and matched demographic data for the entire pediatric SLE cohort abstracted from Space Labs software, using a looser 90th percentile cutoff to distinguish normal versus high BP values. The 90th percentile cutoff is commonly used to distinguish normal versus high BP values from casual BP measurements in populations at high risk for cardiovascular events, such as in individuals with congestive heart failure, diabetes, and chronic kidney disease. Age is in years, BMI = body mass index (Dataset 7: Campbell *et al.*, 2015g). Click here for additional data file. Copyright: © 2015 Campbell JF et al. 2015 Data associated with the article are available under the terms of the Creative Commons Zero "No rights reserved" data waiver (CC0 1.0 Public domain dedication).

## Discussion/Conclusions

This study illustrates the potential benefit for further investigation of ABPM use in characterizing BP patterns in SLE patients. Our results show that pediatric SLE patients have a very high rate of attenuated nocturnal SBP and DBP dipping. This was associated with higher rates of nocturnal HTN (whether isolated or in conjunction with daytime HTN), though with standard ABPM-based definitions, this was not statistically significant. A previous study of subclinical cardiovascular disease in pediatric SLE patients reported similar findings with 14 of 21 patients having attenuated nocturnal dipping and higher nocturnal BPs when compared to daytime BPs ( Canpolat *et al.*, 2013). However the prior study’s primary focus was cardiovascular risk. There was no control group, and the relationships between ABPM findings and clinical characteristics tested were limited to echocardiogram findings. The small number of studies using ABPMs to characterize BPs in other pediatric chronic illnesses, such as sickle cell disease, has been revealing. This is the first study to investigate the relationship between BP characteristics on ABPM and clinical characteristics in the pediatric SLE population.

SLE patients are at increased risk for death and cardiovascular disease is a leading cause of mortality in this population. This is related to both traditional and non-traditional risk factors in adult patients ( Knight & Kaplan, 2013). In a study of 94 adults with SLE, correlations were noted between intima-medial thickness and clinical disease activity scores ( Oryoji *et al.*, 2013). Similarly, in a separate study of 64 adults with SLE and nephritis in complete remission, 53% were hypertensive ( Shaharir *et al.*, 2015), and the risk factors identified included disease duration (odds ratio (OR) 1.06), longer duration interval to achieving remission (OR 1.10), and the number of disease relapses (OR 2.5). There were no associations between histological classes of nephritis, body mass index, or waist circumference. A study of 51 children with SLE also demonstrated that functional and morphological cardiovascular changes were independent of traditional risk factors such as daytime HTN, hypertriglyceridemia, diabetes, and chronic kidney disease ( Sozeri *et al.*, 2013). In SLE, these changes in arterial stiffness, intima-media thickness, and LV mass ( Canpolat *et al.*, 2013; Oryoji *et al.*, 2013; Sozeri *et al.*, 2013) are likely to be almost entirely secondary to non-traditional factors, such as disease-related mechanisms like enhanced apoptosis, aPL antibodies, circulating immune complexes, and vasculitis.

Therefore it is important to understand the BP characteristics of these patients, particularly the nocturnal BP patterns, as our study shows that they differ from the general population. There was no effect on this altered ABPM blood pressure pattern in our cohort attributable to medication usage, complement cascade activation and hypocomplementemia, or titers of ANA, aPL antibodies, or anti-dsDNA antibodies. Future studies of ABPM testing in SLE populations can be designed to further assess clinical parameters such as degrees of systemic inflammation, interferon versus neutrophil signatures, or endothelial cell dysfunction, in order to try to understand the possible mechanisms for elevated nocturnal BPs and attenuated nocturnal dipping in SLE patients.

Based on our study, the duration of disease did not seem to play a role in the attenuated dipping, as this pattern was seen even within the first year after SLE diagnosis. Since 90% of SLE patients had attenuated dipping, compared with only 60% of patients meeting criteria for diagnosis of nocturnal hypertension, one might conclude that attenuated nocturnal BP dipping is an earlier change that progresses to nocturnal HTN in the setting of SLE. However, the SLE patient without attenuated dipping did have nocturnal hypertension. Therefore, it is more likely that the disease process in SLE leads to cardiovascular changes sufficient to cause elevated nighttime BP very early in the disease course. If nocturnal HTN or attenuated BP dipping turn out to be pathogenic in SLE, then monitoring for HTN solely with casual daytime clinic measurements may postpone possible interventions that could potentially reduce the increased cardiovascular risk faced by these patients.

One limitation of this study is the small number of patients in the SLE group and the resulting low power. Several findings trended toward significance and might become statistically significant with a larger study population. Second, most patients were taking prednisone at time of ABPM, and our study is unable to address the role of glucocorticoids versus the role of SLE on rhythmicity of BP. The only other study of ABPM in SLE involving a cohort of 10 adult patients from Japan looked before and after prednisolone treatment ( Imai *et al.*, 1989). These patients received a mean dose of 40 ± 17 mg/day for 58 ± 19 days between testing. The results showed an attenuated nocturnal dipping after treatment that was not identified beforehand. Therefore, the hypothalamic-pituitary-adrenal axis may also have impacted the results in our pediatric cohort. A previous study of ABPM in patients with Cushing’s Syndrome has shown attenuated nocturnal dipping, compared to control subjects and patients with essential HTN ( Piovesan *et al.*, 1990). Moreover, a study of 11 healthy males showed a less pronounced fall in nocturnal SBP after a 4 day course of oral prednisolone ( Ivarsen *et al.*, 1995). In a Czech cohort of 60 adults with rheumatoid arthritis, those taking only prednisone and nonsteroidal anti-inflammatories were more often non-dippers, but those taking nonsteroidals, prednisone and methotrexate more often showed excessive nocturnal dipping ( Rihacek *et al.*, 2009). Since the pediatric SLE patients described here were taking additional immunomodulatory agents as well as prednisone at time of ABPM, distinguishing the effects of disease and treatment would be difficult.

Although there were a limited number of statistically significant findings, strict inclusion of only SLE patients with prehypertension or stage 1 hypertension, without active nephritis, and most without anti-hypertensive medication, provided for a valid comparison between children with SLE and non-lupus controls. Our study does show that nocturnal HTN and attenuated nocturnal dipping do occur more frequently in pediatric SLE patients than in the non-SLE population. Further research is warranted regarding the association of these findings with other clinical characteristics.

## Data availability

The data referenced by this article are under copyright with the following copyright statement: Copyright: © 2015 Campbell JF et al.

Data associated with the article are available under the terms of the Creative Commons Zero "No rights reserved" data waiver (CC0 1.0 Public domain dedication). 

*F1000Research*: Dataset 1. Raw ABPM Data for pSLE Cohort, 10.5256/f1000research.6532.d49239 ( Campbell *et al.*, 2015a).

*F1000Research*: Dataset 2. Raw ABPM Data for non-SLE Cohort, 10.5256/f1000research.6532.d49240 ( Campbell *et al.*, 2015b).

*F1000Research*: Dataset 3. Raw Data: Sensitivity Analysis: non-SLE greater than 50 successful readings, 10.5256/f1000research.6532.d49241 ( Campbell *et al.*, 2015c).

*F1000Research*: Dataset 4. Raw Data: sensitivity analysis: non-SLE, greater than 75 percent successful, 10.5256/f1000research.6532.d49242 ( Campbell *et al.*, 2015d).

*F1000Research*: Dataset 5. Raw Data: Sensitivity Analysis: pSLE greater than 50 successful readings, 10.5256/f1000research.6532.d49257 ( Campbell *et al.*, 2015e).

*F1000Research*: Dataset 6. Raw Data: Sensitivity analysis: pSLE greater than 75 percent successful, 10.5256/f1000research.6532.d49258 ( Campbell *et al.*, 2015f).

*F1000Research*: Dataset 7. Raw Data: Sensitivity analysis: pSLE using 90th percentile, 10.5256/f1000research.6532.d49259 ( Campbell *et al.*, 2015g).

## Consent

A waiver of consent was obtained from the Institutional Review Board for this study.
