# Supplementary material for: Nocturnal Hypertension and Attenuated Nocturnal Blood Pressure Dipping is Common in Pediatric Lupus
Source: F1000Res. 2015 Nov 23;4:164. Originally published 2015 Jun 23. [Version 2] doi: 10.12688/f1000research.6532.2 (PMC4654458; doi:10.12688/f1000research.6532.2)
Supplement: Peer review history

# Peer review: Referee response for version 2

**DOI:** 10.5256/f1000research.8033.r10970

**Published:** 2015-11-30

**Authors:** Rene VanDeVoorde

**Affiliations:** 1 Division of Pediatric Nephrology and Hypertension, Cincinnati Children's Hospital Medical Center, Cincinnati, OH, USA

This retrospective study looked at the ambulatory blood pressure monitoring (ABPM) patterns in a small number of pediatric patients with Lupus (SLE) but not nephritis compared to a larger cohort of patients with similar casual blood pressure findings but without SLE. The study design seems appropriate, however the lack of racial/ethnicity information in the comparison group makes it difficult to know if this comparison is entirely appropriate. The Lupus group had 70% African American or Hispanic patients, which are groups whose normal ABPM data are not known and may not be represented by the normative data by Wuhl. Potential matching by age, gender, and ethnicity may have lent more strength to this comparison.

However, the findings of reduced nocturnal dipping in 90% of the SLE group is fairly significant and definitely bears reporting. The authors are quite clear that their small numbers make it difficult to find statistical significant factors within the SLE cohort to explain these findings and that a larger study in this population is merited. The discussion about possible effect of corticosteroids, added in the revision, is one factor that certainly needs to be investigated further. Additionally, the presence of proteinuria in 20% of the SLE patients is somewhat curious as they may have some residual effects from their nephritis which would possibly also manifest with changes in their diurnal BP pattern. However, these are exactly some of the questions should be uncovered in the discussions of a "pilot" study such as this.

I have read this submission. I believe that I have an appropriate level of expertise to confirm that it is of an acceptable scientific standard.

# Peer review: Referee response for version 1

**DOI:** 10.5256/f1000research.7012.r10968

**Published:** 2015-11-16

**Authors:** Coral Hanevold

**Affiliations:** 1 Division of Nephrology, Seattle Children's Hospital, Seattle, WA, USA

Increased risk for cardiovascular disease is a significant cause of long term morbidity of SLE. In this small retrospective study the authors address one of the potential modifiable risk factors for CV disease in this population, hypertension. In this pilot study the authors focused on children and teens with SLE but no renal disease (or nephritis in remission) with prehypertension or stage 1 HTN who had been evaluated with an ABPM. Although the patient numbers are small, the study is strengthened by the use of the non-SLE control group. 

With regard to the methods, findings in the SLE patients were compared to a control group comprised of children undergoing ABPM for evaluation of prehypertension or stage 1 hypertension who did not have SLE (though other details regarding this group are not available). This group was reported to be age matched though it is noted that the mean age of this group was 12.4 years as compared to 14.6 for the SLE group. The mean BMI for the 2 groups was well matched. Unfortunately data for the non-SLE group was limited to information on the ABPM report which was limited to age, sex, height, weight (with BMI calculated from these data). Thus it was not possible to compare race or ethnic group of the control group to the SLE group which was 40% African American and 30% Hispanic.

The authors found that the SLE patients demonstrated a higher prevalence of reduced dipping than the control group (90% vs 26% respectively). Additionally, 60% of SLE patients had nocturnal HTN as compared to 39% of the control group. Due to the small number of patients the authors were not able to demonstrate an association of findings on ABPM with clinical factors such as steroid exposure, disease activity, proteinuria, echocardiograms, etc. Of note all patients with nocturnal hypertension met historical criteria for nephritis even though not active at time of the study. It is possible that these SLE patients had other risk factors for hypertension not addressed here such as positive family history or elevated BMI. Perhaps the authors could comment on the latter point at least. These factors are important given the high prevalence of African American and Hispanic children in their SLE population. Data also showed that SLE patients had a statistically significant lower daytime systolic load as compared to controls while only 50% had elevated nighttime systolic loads and 60% had nocturnal hypertension. Thus it may be that the low daytime systolic load contributed to the finding of attenuated dipping in some of the SLE cohort. 

The authors performed various sensitivity analyses that involved using a lower threshold (90 ^th^ percentile) to define hypertension, use of 30% as cutoff for abnormal load, more stringent requirements for adequacy, and limited non-SLE group to females. These data are somewhat confusing and the authors could consider deleting this section.

The study reported here highlights the need for scrutiny of BPs in SLE patients without active nephritis in whom mild elevations in the office might be attributed to white coat hypertension.  Additionally the high prevalence of abnormalities of nocturnal pressures, often occurring in isolation, indicates that this method of evaluation (ABPM) is crucial in the evaluation of this population. These findings should be the basis for further study in a larger group of patients. The study has significant limitations as mentioned above but the findings are compelling and thus warrant reporting.

I have read this submission. I believe that I have an appropriate level of expertise to confirm that it is of an acceptable scientific standard.

# Peer review: Referee response for version 1

**DOI:** 10.5256/f1000research.7012.r10969

**Published:** 2015-11-03

**Authors:** Joseph Flynn

**Affiliations:** 1 Division of Nephrology, Seattle Children's Hospital, Seattle, WA, USA

The authors present the results of a small retrospective case series of children with systemic lupus studied by Ambulatory Blood Pressure Monitoring (ABPM). Their major finding is that these patients have blunted nocturnal BP dipping, which has been associated with adverse cardiovascular risk. This is a novel finding that while needing to be confirmed in a larger, prospective study, could have major implications for the clinical management of children with SLE.

One issue that requires clarification is how the authors characterize the study population. In several places in the paper they state that the SLE patients don't have nephritis, but in reality, about half had a history of nephritis. The authors should restate the description of the SLE patients to clarify that these children were not currently being treated for active nephritis. There are actually some interesting differences in the children with and without a history of nephritis that could be explored in more detail - for example, the ABPM parameters in Table 2 could be further divided into groups of those with and without a history of nephritis. How would that division then affect the results of the other analyses performed?

Another major issue that requires greater exploration is the effect of prednisone treatment on BP dipping. All of the SLE patients were on prednisone and it is likely that this affected the dipping patterns. This needs to be explicitly acknowledged in the discussion, and some discussion of prior studies (if they exist) exploring the effect of corticosteroids on the circadian variation of BP needs to be added.

Minor suggestions:

- add the number of subjects to the abstract (it only gives the number of non-SLE controls)
- shorten the introduction by 50% (first 2 paragraphs and the first 2/3 of the third paragraph could be deleted)
- I don't think the sensitivity analysis adds anything; this could probably be deleted as well
- it is unlikely that specific laboratory findings would affect BP outcomes, could some of this information be removed as well?

I have read this submission. I believe that I have an appropriate level of expertise to confirm that it is of an acceptable scientific standard, however I have significant reservations, as outlined above.

# Response

**Published:** 2015-11-17

**Authors:** Scott Wenderfer

**Affiliations:** Baylor College of Medicine, USA

The authors appreciate the detailed and thorough reviews performed by all of the referees, including Dr. Flynn. We have submitted a modified and improved version of the manuscript addressing the comments and suggestions, as summarized in our response here.

*One issue that requires clarification is the study population. In several places in the paper the authors state that the SLE patients don't have nephritis; but in reality, about half had a history of nephritis. The authors should restate the description of the SLE patients to clarify that these children were not currently being treated for active nephritis.*

- Done (modified the Study Population section of the Methods as well as in the Patient and Clinical Characteristics section of the Results)

*There are actually some interesting differences in the children with and without a history of nephritis that could be explored in more detail - the ABPM parameters in Table 2 could be further divided into groups of those with and without a history of nephritis.*

- We thank the referee for this suggestion. Table 2 has been modified to sub-divide our pSLE cohort into the 4 non-renal pSLE patients and 6 patients with a history of lupus nephritis which was in remission and not being treated at the time of ABPM. The table now shows that nighttime SBP and DBP loads were higher in SLE patients with a history of nephritis than in non-renal lupus patients, even though the nephritis was in remission at time of ABPM. Moreover, it shows that all SLE patients with nocturnal HTN had a history of nephritis.

*Another major issue that requires greater exploration is the effect of prednisone treatment on BP dipping.*

- We agree that the effect of pharmacologic therapy in patients with pSLE is a confounding factor in the interpretation of ABPM data in our cohort of patients, and we agree that there may indeed be an effect of the prednisone on the attenuated nocturnal dipping. We have added a paragraph to the discussion describing four studies that support this notion. Unfortunately, our cohort of patients is too small to distinguish between disease-specific and treatment-specific effects. Nonetheless, as long-term prednisone use in patients with SLE is nearly universal, our results emphasize the need to consider nocturnal HTN in the management of these patients.

*Minor suggestion: add the number of subjects to the abstract.*

- Done (line 9 of the abstract)

# Peer review: Referee response for version 1

**DOI:** 10.5256/f1000research.7012.r9811

**Published:** 2015-08-17

**Authors:** Joyce Popoola

**Affiliations:** 1 Department of Renal Medicine and Transplantation, St George, London, UK

This is a retrospective study looking at nocturnal blood pressure dips in paediatric patients with lupus. It is descriptive in nature and looks at small numbers as it is a single centre review of a relatively rare condition in the paediatric population. Though the paper shows a statistically significant nocturnal dip in the systolic and diastolic blood pressure compared to the controls it is unable to dissect out demographics or other characteristics that could be used to explain this because of the small numbers. This is an important question as blood pressure will have an impact on long term effect of this condition and potential development of renal disease beyond lupus nephritis. The paper offers no opportunity for mechanistic explanations for the observations because of the retrospective nature and small numbers as recognised by the authors.

The statistics are also limited for the same reasons. I feel the paper should be published however to highlight the issue and also to provide a platform for the authors to raise awareness to the issue and enable a multi centre prospective study of the same question dissecting out demographics (age stratification, ethnicity), other co-morbidities (this would be more of an issue in an adult population) and to test out the mechanisms of this process potentially examining vascular resistance and flow mediated dilatation. It would also be of interest to see the effect of treatment on this phenomenon in a longitudinal fashion.

I have read this submission. I believe that I have an appropriate level of expertise to confirm that it is of an acceptable scientific standard.
